# TNF-α-Producing *Cryptococcus neoformans* Exerts Protective Effects on Host Defenses in Murine Pulmonary Cryptococcosis

**DOI:** 10.3389/fimmu.2019.01725

**Published:** 2019-07-26

**Authors:** Zhenzong Fa, Jintao Xu, Jiu Yi, Junjun Sang, Weihua Pan, Qun Xie, Runping Yang, Wei Fang, Wanqing Liao, Michal A. Olszewski

**Affiliations:** ^1^Division of Pulmonary and Critical Care Medicine, Department of Internal Medicine, University of Michigan Health System, Ann Arbor, MI, United States; ^2^Shanghai Key Laboratory of Molecular Medical Mycology, Department of Dermatology, Changzheng Hospital, Naval Medical University, Shanghai, China; ^3^Department of Dermatology, The Sixth Medical Center of PLA General Hospital, Beijing, China; ^4^Ann Arbor VA Health System, Department of Veterans Affairs Health System, Ann Arbor, MI, United States; ^5^Department of Anesthesiology, The Fourth Medical Center of PLA General Hospital, Beijing, China

**Keywords:** cryptococcosis, TNF-α, host defense, TNF-α-producing *C. neoformans*, Th1/Th2 balance

## Abstract

Tumor necrosis factor alpha (TNF-α) plays a critical role in the control of cryptococcal infection, and its insufficiency promotes cryptococcal persistence. To explore the therapeutic potential of TNF-α supplementation as a booster of host anti-cryptococcal responses, we engineered a *C. neoformans* strain expressing murine TNF-α. Using a murine model of pulmonary cryptococcosis, we demonstrated that TNF-α-producing *C. neoformans* strain enhances protective elements of host response including preferential T-cell accumulation and improved Th1/Th2 cytokine balance, diminished pulmonary eosinophilia and alternative activation of lung macrophages at the adaptive phase of infection compared to wild type strain-infected mice. Furthermore, TNF-α expression by *C. neoformans* enhanced the fungicidal activity of macrophages *in vitro*. Finally, mice infected with the TNF-α-producing *C. neoformans* strain showed improved fungal control and considerably prolonged survival compared to wild type strain-infected mice, but could not induce sterilizing immunity. Taken together, our results support that TNF-α expression by an engineered *C. neoformans* strain while insufficient to drive complete immune protection, strongly enhanced protective responses during primary cryptococcal infection.

## Introduction

The fungal pathogen *Cryptococcus neoformans* causes substantial morbidity and mortality worldwide, accounting for an estimated 220,000 cases of cryptococcal meningoencephalitis and 180,000 deaths among people with HIV/AIDS each year (1–4). The current treatment options for cryptococcal infections are limited and often unsuccessful due to the high toxicity and the poor blood-brain barrier permeability of the antifungal drugs (5, 6). Acute mortality for cryptococcal infections is up to 30%, and many surviving patients may develop permanent disabilities (7–9). Proper containment of *C. neoformans* by host requires the development of optimal innate and adaptive immune responses (2). Murine models have shown that protective anti-cryptococcal immunity depends on the generation of strong Th1/Th17 T-cell responses, which in turn, promote classical activation of macrophages and effective containment of *C. neoformans*. In contrast, Th2 response facilitates eosinophil recruitment and alternative activation of macrophages, which promote intracellular survival and growth of *C. neoformans* during infection (2, 10–13). Apart from the effector functions during the adaptive response, macrophages, and dendritic cells (DC) play active roles in the early/innate defenses. They ingest *C. neoformans*, providing first-line of anti-fungal containment in the infected lungs. Through fungal antigen processing, presentation, and concurrent inflammatory cytokine production, they initiate and polarize the development of T-cell responses (10, 14).

Tumor necrosis factor alpha (TNF-α), a major player during innate and adaptive responses, has been shown to play critical roles in host defense against *C. neoformans* infection. Early TNF-α production in a murine model infected with a moderately virulent *C. neoformans* strain 52D is required for the development of protective Th1/Th17 immune responses and subsequent fungal containment (15–17). Recent studies showed that early TNF-α induction contributes to DC classical activation, followed by the generation of protective adaptive responses during the efferent phase of *C. neoformans* infection (17, 18). Furthermore, the enhanced risks for cryptococcal infection in individuals undergoing anti-TNF-α therapies for sepsis, cancer, and autoimmune disorders provided strong evidence that this cytokine also plays an important role in mediating host defense against *C. neoformans* infection in human (18–20). However, highly virulent *C. neoformans* strains are endowed with a capsule that can attenuate host production of TNF-α (21, 22). During infection, mice infected with a highly virulent *C. neoformans* strain show significantly lower TNF-α expression in the lungs compared to mice infected with less virulent *C. neoformans* strain (23). Thus, highly virulent *C. neoformans* could evade host anti-cryptococcal immunity by inducing minimal TNF-α response.

Considering this broad significance of TNF-α and the inhibitory effect of highly virulent *C. neoformans* on its production, we hypothesized that therapeutic delivery of TNF-α could be a mean for augmenting host immune defenses against *C. neoformans* infections. Studies have shown that *C. neoformans* strain constructed to constitutively produce and secrete low of murine IFN-γ successfully induced protection against primary and second pulmonary challenges with a pathogenic *C. neoformans* strain H99 (24). In this study, we generated analogous *C. neoformans* strain engineered to express murine TNF-α with a goal to stimulate a protective response against cryptococcal infection. These studies have provided evidence that TNF-α expression by highly virulent *C. neoformans* strain can enhance protective responses and suggest that adjuvant TNF-α therapy may represent a viable approach supporting treatment of *C. neoformans* infection.

## Materials and Methods

### Ethics Statement

These studies were performed in compliance with the protocols approved by the institutional R&D, animal studies, and research safety committees of the VA Ann Arbor Health System. Ann Arbor VA Animal Studies Committee approved these studies (protocol number 0512-025) in strict accordance with the recommendations in the Guide for the Care and Use of Laboratory Animals of the National Institutes of Health. All manipulations involving live mice were performed under general anesthesia, and endpoint criteria were used for survival experiments to humanely euthanize moribund animals. All efforts were made to ensure proper care for the animals and to minimize suffering.

### Mice

Female Balb/c mice (Jackson Laboratory, Bar Harbor, ME, USA) were housed under specific-pathogen-free (SPF) conditions at the Ann Arbor Veterans Affairs Medical Center. Animals were 8–12 weeks old at the time of infection. Mice were humanely euthanized using CO_2_ inhalation at the time of data collection.

### C. neoformans

For H99-α construction, an expression construct containing cryptococcal *ACTIN* promoter and murine TNF-α cDNA sequences was transformed into *C. neoformans* serotype A strain H99 by a biolistic method as described previously (25, 26). For inoculation, *C. neoformans* H99-α and wild-type (WT) H99 strain were grown for 4 days at 37°C using Sabouraud dextrose broth (1% Neopeptone, 2% dextrose; Difco, Detroit, MI, USA). The cultures were centrifuged and washed with non-pyrogenic saline (Travenol, Deerfield, IL, USA). Cells were counted via hemocytometer and diluted to 3.3 ×10^5^ yeast/ml in sterile non-pyrogenic saline. Serial dilutions of the *C. neoformans* suspension were plated on Sabouraud dextrose agar (SDA) to confirm the number of viable fungi in the inoculum.

### Transformation of *C. neoformans* With a Murine TNF-α Construct

In order to avoid the disruption of exogenous DNA constructs on adjacent genes, we remodeled the plasmid vector (pCH233) to facilitate the integration of the TNF-α cassette into a small gene-free region (“safe haven”) as previously described (27). The “safe haven” region was PCR amplified from *C. neoformans* genomic DNA as two fragments using primers ZF0001&ZF0002 and ZF0003&ZF0004 and the nourseothricin resistance marker NAT was amplified from pCH233 using primers ZF0005&ZF0006 to give a 1.9 kb product. The three fragments were joined to generate a 3.5 kb product via overlap PCR using primers ZF0001 and ZF0004, purified, and then infused with purified vector sequence (amplified from the plasmid pCH233 by PCR using primers ZF0011&ZF0012 containing SfiI sites on both 5′ and 3′ flanking regions) by pEASY-Uni Seamless Cloning and Assembly Kit (Transgen Biotech). The new vector pCH235 was transformed into *E. coli*, and confirmed by diagnostic PCR (using primers ZF0001 and ZF0005) and sequencing.

**Figure 1:**
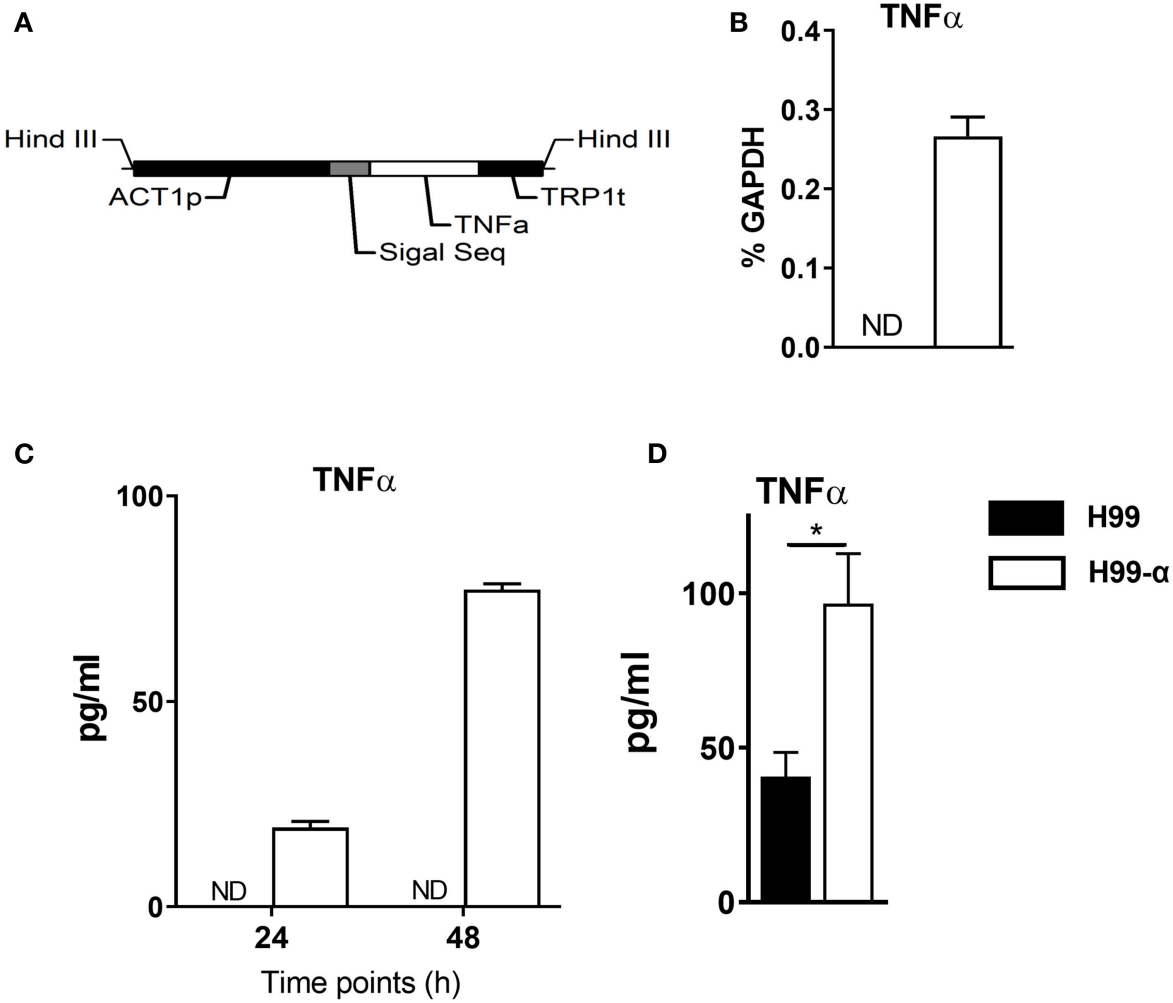
Construction of the murine TNF-α-producing transgenic *C. neoformans* strain. **(A)** expression construct containing *C. neoformans ACTIN* promoter and murine TNF-α cDNA sequences. **(B,C)** TNF-α expression by the transgenic strain H99-α was detected by RT-qPCR or by ELISA in the culture media at 37°C. **(D)** TNF-α level in the lung homogenates of mice infected with H99-α or H99 intratracheally at 48 h post-infection (Results represent mean ± SEM; ^*^P < 0.05).

The *ACTIN* promoter region and TRP1 terminator region were amplified from the vector pCH233 by PCR using High Fidelity DNA Polymerase PrimeSTAR (Takara) using primers ZF0007&ZF0008 and ZF0009&ZF0010, respectively. The whole encoding region was synthesized by Thermo Fisher Scientific, including the signal sequence of the phospholipase B (PLB) gene from genomic DNA derived from *C. neoformans* strain H99 and Murine TNF-α cDNA (soluble form, GenBank No. AK155964.1). Above fragments were then infused into a murine TNF-α construct (1.8 kb, Figure 1A) by overlap PCR using primers ZF0007 and ZF0010 containing each HindIII site on both upstream and downstream flanking regions. The TNF-α construct was purified and then infused with linearized vector pCH235 (digested with restriction endonuclease HindIII). The new plasmid pCH-TNFA was digested with SfiI to generate a 5.3 kb product (TNF-α integration cassette) containing the safe haven, murine TNF-α construct and *NAT* gene. The final product was purified and then transformed into the *C. neoformans* genome via biolistic delivery system as previously described (26). Transformants were selected on YPD media plus 100 μg/ml nourseothricin (clonNAT; Werner Bioagents, Jena, Germany), and confirmed by Southern blot, colony PCR and real-time PCR. Culture supernatant of TNF-α secreting and WT *C. neoformans* strains were evaluated for TNF-α protein production by enzyme-linked immunosorbent assay (ELISA) as described below. Primers used in this study are listed in Table 1.

**Table 1:** Primers used in this study.

**Primer**	**Sequence (5^**′**^-3^**′**^)**	**Function**
ZF0001	CAATTTCACA*GGCCATTTAGGCC*CCAATCTCGCTGACTTTCTA	Amplification of “safe haven” region
ZF0002	CATAGCTGTTTCCTGCCAATGAAGCACGCTCAAA	Amplification of “safe haven” region
ZF0003	TGGCCGTCGTTTTACCTTTGGCCAATCTCTTTCAC	Amplification of “safe haven” region
ZF0004	TCACGACGTT*GGCCATATAGGCC*TTTCCATCAGTAACATCGGG	Amplification of “safe haven” region
ZF0005	GTAAAACGACGGCCAG	Amplification of *NAT* gene
ZF0006	CAGGAAACAGCTATGAC	Amplification of *NAT* gene
ZF0007	TGATTACGCC*AAGCTT*GCTGCGAGGATGTGAGCT	Amplification of ACT1 promoter region
ZF0008	GTAGCCGTGGCGATTGACATAGACATGTTGGGCGAGT	Amplification of ACT1 promoter region
ZF0009	CTTTGGAGTCATTGCTCTGTGAGTGAAGGCGGTAAGGGGTT	Amplification of TRP1 terminator region
ZF0010	GCTCGGTACC*AAGCTT*GGTTTATCTGTATTAACACGGA	Amplification of TRP1 terminator region
ZF0011	AC*GGCCTATATGGCC*AACGTCGTGACTGGGAA	Constructing of plasmid (pCH234)
ZF0012	TGGGCCTAAATGGCCTGTGAAATTGTTATCCGCTC	Constructing of plasmid (pCH234)
GAPDH-F	TATGTCGTGGAGTCTACTGGT	qPCR
GAPDH-R	GAGTTGTCATATTTCTCGTGG	qPCR
ARG1-F	CAGAAGAATGGAAGAGTCAG	qPCR
ARG1-R	CAGATATGCAGGGAGTCACC	qPCR
iNOS-F	GCATTGGAAGTGAAGCGTTTC	qPCR
iNOS-R	GGCAGCCTGTGAGACCTTTG	qPCR

### Capsule Size Measurement

This method is referenced to previous studies with modification (28). Briefly, *C. neoformans* was incubated at 37°C in YPD broth with shaking for 24 h. Wash with PBS three times. Count the cells and resuspend at 2 × 10^5^ cells/2 mL in Dulbecco's minimal essential media (Gibco) following incubation at 37°C and 5% CO_2_ for 3 days. Cells were harvested by centrifuging. Remove all but 5–10 μL supernatant. Carefully resuspend the cells and stain with India ink on a glass microscope slide. Cover and seal. Images (>50 cells/strain) were acquired using a Zeiss light microscopy (ZEISS, AXIO) and diameters were measured using VistarImage software (x64,4.0.10289.20171114).

### Intratracheal Inoculation of *C. neoformans*

Mice were anesthetized via intraperitoneal injection of ketamine (100 mg/kg body weight) plus xylazine (6.8 mg/kg) and were restrained on a foam plate. A small incision was made through the skin and the underlying salivary glands and muscles were separated to expose the trachea. Total 10^4^ CFU of *C. neoformans* (3.3 × 10^5^ yeast/ml in 30 μl) were intratracheally injected into the lungs. Culturing and trypan blue staining of the inoculated solutions were used to confirm the viability of yeast. After inoculation, the skin was closed with cyanoacrylate adhesive and the mice were monitored during recovery from the anesthesia.

### *In vitro* Virulence Assays

To evaluate growth in liquid YPD media at 37°C, H99-α and H99 culture were serially diluted and plated on SDA for 3 days. To evaluate growth on agar plates under different conditions, the yeast H99-α and H99 cells were firstly cultured to saturation in YPD broth at 30°C, harvested and washed twice with 1 × PBS buffer. The yeast cells were serially diluted and spotted on different stress media. For the oxidative and NO stress tests, the cells were incubated on Yeast Nitrogen Base (YNB) agar containing 2 mM H_2_O_2_ or 0.75 mM NaNO2 (pH 4.0). To evaluate the response to high salt and osmotic stresses, 1.5 M NaCl or 1.5 M sorbitol was added to the YPD agar. To analyze melanin production, *C. neoformans* strains were spotted on L-DOPA and caffeic acid medium and incubated for 5 days at 30°C (29). For capsule production, fungal cells were incubated on DMEM medium for 3 days in the presence of 5% CO_2_ at 37°C and the capsule was stained with India ink. For urease assay, strains were cultured onto Christiansen's urea agar at 30°C for 24 h.

### Macrophage Killing Assays

Bone marrow-derived macrophages (BMMs) were generated as previously described (30). To evaluate the fungicidal activity of BMMs to H99 and H99-α, 1 × 10^5^ BMMs were seeded on a 96-well plate and infected with *C. neoformans* yeast (1 × 10^4^) with or without IFN-γ (100 ng/ml). After a 24-h incubation, the total yeasts were collected by lysing BMMs with 0.5% SDS and enumerated by plating onto SDA plates. Colony values in individual groups were compared with culture wells without BMMs and expressed as a percentage of growth inhibition.

### Leukocytes Isolation From the Lungs

Mice were euthanized with CO_2_ and then perfused with PBS. The lungs were aseptically removed, minced with scissors, transferred to GentleMACs C tubes containing 5 ml of digestion buffer [RPMI 1640, 5% FBS, penicillin, and streptomycin; 1 mg/ml collagenase A (Roche Diagnostics); and 30 μg/ml DNase I]. After incubation at 37°C for 35 min, the cell suspension and tissue fragments were further dispersed on a GentleMACs homogenizer (Miltenyi). Erythrocytes in the cell pellets were lysed by addition of 3 ml NH4Cl buffer (0.829% NH_4_Cl, 0.1% KHCO_3_, and 0.0372% Na_2_EDTA, pH 7.4) for 3 min. Cells were re-suspended and subjected to syringe dispersion and filtered through a sterile 100-μm nylon screen (Nitex). The filtrate was centrifuged for 30 min at 1,500 × g with no brake in the presence of 20% Percoll (Sigma) to separate leukocytes from cell debris and epithelial cells. Total cell numbers were determined by counting live cells on a hemocytometer with trypan blue. Percentages of leukocyte subsets were calculated by multiplication of total cell number recovered from dispersed lungs multiplied by percentages of each subset defined via flow cytometric analysis using a set of specific markers, listed below.

For enrichment of adherent macrophages from the lungs, digested lung leukocytes were incubated for 90-min on tissue culture plastic. Non-adherent cells were rinsed away as previously described (31). Remaining macrophages were collected in Trizol reagent.

### Ag-Specific Cytokine Production by Lung Leukocytes

Isolated lung leukocytes were cultured in media with heat-killed H99 in a ratio of 1:10 in 6-well plates with 2 ml complete RPMI 1640 medium at 37°C and 5% CO_2_ for 48 h. Supernatants were stored and analyzed using a LEGENDplex cytometric bead array (CBA) kit (BioLegend, San Diego, CA, USA) following the manufacturer's specifications and read on an LSRII flow cytometer (Becton, Dickinson Immunocytometry Systems, Mountain View, CA, USA). The analysis was performed using BioLegend's LEGENDplex software.

### RT-qPCR

Total RNA was prepared using TRIzol reagent (Invitrogen), and the first-strand cDNA was synthesized using Reverse Transcription Kit (Qiagen, Hilden, Germany) according to the manufacturer's instructions. Relative gene expression was quantified with SYBR green-based detection (Alkali Scientific) using a light cycler96 system (Roche) according to the manufacturer's protocols. Relative gene expression was normalized to *GAPDH* mRNA using the 2^−Δ*Ct*^ methods. For some experiments, β*-*actin and 18S rRNA were also used as additional housekeeping genes to validate results calculated relative to GAPDH.

### Flow Cytometric Analysis

Ab cell staining was performed as previously described (32). For staining, cells were isolated, washed in PBS, and then stained with Live Dead Fixable Aqua (Life Technologies) for 30 min. Cells were then stained with antibodies (see below), washed, and fixed in 2% formaldehyde. Data were collected on a FACS LSR2 flow cytometer using FACSDiva software (Becton Dickinson Immunocytometry Systems, Mountain View, CA, USA) and analyzed using FlowJo software (Tree Star, San Carlos, CA, USA).

The following gating strategy was used to identify leukocyte subsets in the lungs. First, consecutive gates identified singlets, live cells, and CD45+ leukocytes. Next, a series of selective gates were used to identify neutrophils (CD11b^+^Ly6G^+^); eosinophils (SSC^high^CD11c^low^/SiglecF^+^); Alveolar macrophages (CD11c^high^/SiglecF^+^); Ly6C^high^ monocytes (CD11c^−^/CD11b^+^/Ly6C^high^), and DC (CD11c^+^MHCII^high^), Thereafter, DC were further separated into moDC, CD11b^+^ DC and CD103^+^ DC as follows: moDC were gated as CD11c^+^MHCII^high^CD64^+^ cells, then remaining CD11c^+^MHCII^high^CD64^−^ cells were further divided into CD11b^+^ DC and CD103^+^ DC based on the expression of SIRPα and XCR1, respectively. Isotype control antibodies were used to set gates for positive events in all flow cytometric analyses.

### *In vitro* Macrophage Stimulation and Nitrite Production Assay

1 × 10^5^ BMMs were seeded on a 96-well plate and infected with *C. neoformans* yeast (1 × 10^4^). After a 24-h (or 48-h) incubation, the supernatant was centrifuged for nitrite concentration test. Briefly, 100-μL Griess reagent (0.1% naphthyl ethylenediamine in water and 1% sulfanilamide in 5% orthophosphoric acid; Sigma, Aldrich, St. Louis, MO) was added to 100-μL experimental supernatants or 100-μL sodium nitrite standard (0–100 μmol/L). The mixture was incubated at room temperature for 10 min, and the absorbance was measured at 550 nm. Nitrite concentrations of experimental samples were determined by reference to a standard curve constructed in parallel. BMM cells were lysed using TRIzol reagent (Invitrogen), following RNA extraction and RT-PCR as described above.

### Calculations and Statistics

Values are reported as the arithmetic mean ± standard error of the mean. Student *t*-test or two-way ANOVA with a Bonferroni *post-hoc* test were used for comparisons of individual means. All experiments were repeated at least three times. Statistical calculations were performed using GraphPad Prism version 6.00. Means with *p* values <0.05 were considered significantly different.

## Results

### Transgenic Construct *C. neoformans* H99-α Produces Murine TNF-α

We generated an expression construct in which *C. neoformans ACTIN* promoter was used to drive the expression of the murine TNF-α cDNA sequence. The signal sequence of cryptococcal *PLB1* gene was also included in the expression construct to ensure extracellular secretion of TNF-α (Figure 1A). The expression construct was transformed into the WT *C. neoformans* strain H99, and single insertion of murine TNFα sequences into the genome of transformant cells was confirmed by PCRs and Southern blot. One of the successfully transformed strains, designated H99-α, was selected for further study. While no TNF-α expression could be detected in the WT H99 strain, H99-α showed detectable expression of TNF-α at both transcription and protein level when cultured in liquid SDB media at 37°C (Figures 1B,C) To determine *in vivo* TNF-α production during infection, mice were intratracheally infected with H99-α and H99, respectively. Mice infected with H99-α showed significantly greater TNF-α production in the lung homogenates at 48 h post-inoculation compared to H99-infected mice (Figure 1D).

### Expression of the TNF-α Has No Effect on Fungal Fitness and Expression of Classical Virulence Factors by *C. neoformans in vitro*

**Figure 2:**
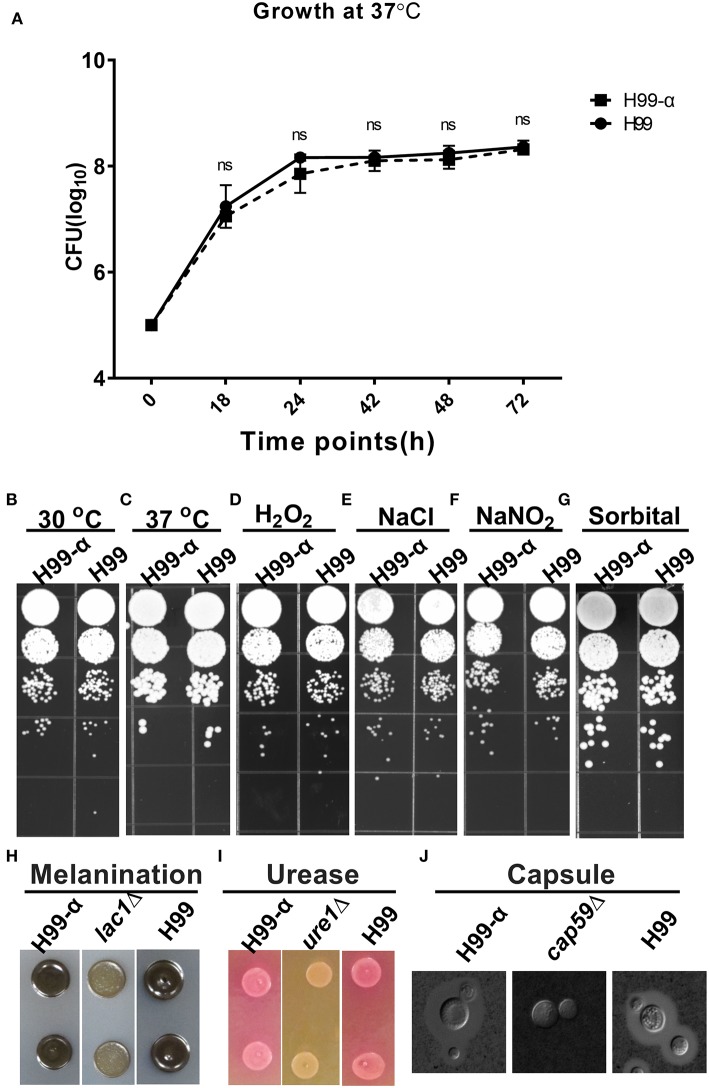
Expression of the murine TNF-α has no effect on cryptococcal fitness and major virulence factor production *in vitro*. **(A)** Growth curve of H99-α and H99 in liquid SDB media at 37°C. **(B)** Growth of H99-α and H99 on SD agar at 30°C **(B)** and 37°C **(C)**, and agar plates containing H_2_O_2_ **(D)**, NaCl **(E)**, NaNO_2_ **(F)**, sorbitol **(G)**. H99 and H99-α melanin production **(H)**, urease activity **(I)**, and capsule formation **(J)** with negative controls *lac1*Δ, *ure1*Δ, and *cap59*Δ for **(H–J)**, respectively. Note that TNF-α expression by H99-α has not affected cryptococcal fitness or the expression of major virulence factor (Results represent mean ± SEM; ns, no Significance).

We next evaluated whether the expression of TNF-α affected cryptococcal fitness and virulence factor expression *in vitro*. H99-α strain showed no significant differences in growth rate in both liquid culture media at 37°C (Figure 2A) or on agar plates at 30°C or 37°C (Figures 2B,C) compared to WT H99 strain, demonstrating intact thermotolerance. H99-α also exhibited similar sensitivity to oxidative, hyperosmotic, and high salt stresses to that of the WT H99 strain (Figures 2D–G). Melanin (Figure 2H), urease (Figure 2I), and capsule production (Figure 2J and Figure S1) were also observed to be the same in H99-α as in the WT H99. Thus, the H99-α strain had no defects in the *in vitro* phenotypes commonly associated with cryptococcal fitness and expression of virulence factors.

### Mice Showed Improved Fungal Control and Extended Survival Time During Pulmonary Infection With H99-α

**Figure 3:**
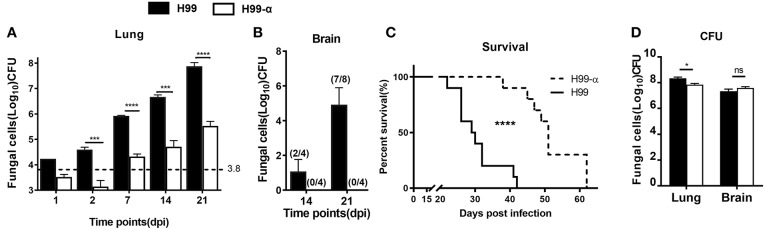
Induction of TNF-α by the transgenic strain improve survival and fungal containment in comparison with the WT strain. BALB/c mice were infected with an intratracheal inoculation of 10^4^ cells of H99-α or WT *C. neoformans* strain H99. Fungal burden from lungs **(A)** and brains **(B)** were quantified at different time points. **(C)** Mice survival was evaluated. *N* = 10 mice per group. **(D)** H99-α infected mice showed comparable fungal burden in both lungs and brains with H99 at time of death. BALB/c mice were infected with an intratracheal inoculation of 104 cells of H99-α or WT *C. neoformans* strain H99. Fungal burden from lungs and brains were quantified when mice dead (weight loss ≥ 20%). Note that the number on the graph **(A)** indicates in the initial inoculation dose of fungal organisms. The numbers on the graph **(B)** indicate the number of mice which are detected with fungal dissemination out of the group size (Results represent mean ± SEM; ^*^P < 0.05; ^***^P < 0.001; ^****^P < 0.0001).

Having determined that H99-α, apart from the production of murine TNFα, showed all the *in vitro*-features of the WT H99 strain, we next evaluated how TNF-α secreted by the transgenic strain affected host responses during *C. neoformans* infection. BALB/c mice were inoculated with a dose of 10^4^ CFU of either H99-α or H99 intratracheally and fungal burdens were evaluated at different time points in the lungs and brains. Mice infected with H99-α exhibited a substantial decrease (100-fold) in fungal burden in the lungs, starting from 2 days post infection (dpi) and continuing through at least 3-weeks (Figure 3A). Fungal CNS dissemination was also ablated in H99-α infected mice at 14 and 21 dpi (Figure 3B). Furthermore, H99-α infected mice showed a much longer survival time with a median survival time of 51 days, compared with 27 days in H99 infected group (Figure 3C). We further evaluated the fungal burden in the lungs and brains of mice from both groups when mice succumbed to infection. We found that both groups of mice showed overwhelmingly high lung fungal burdens and a profound level of CNS dissemination at the time of death (Figure 3D). While pulmonary fungal load in H99-α-infected mice was slightly but significantly lower compared to the H99-infected group, there was no difference CNS burden between groups at mice harvested at the end-stage of the disease (Figure 3D). Taken together, our results suggested that murine TNF-α production by highly virulent of *C. neoformans* H99 can enhance host anti-cryptococcal defenses and fungal containment, but ultimately cannot oppose uncontrolled expansion and dissemination of cryptococcal organism in the infected host.

### Fungal Containment of TNF-α Producing Transgenic Strain Limits Lung Pathology in the Infected Lungs

**Figure 4:**
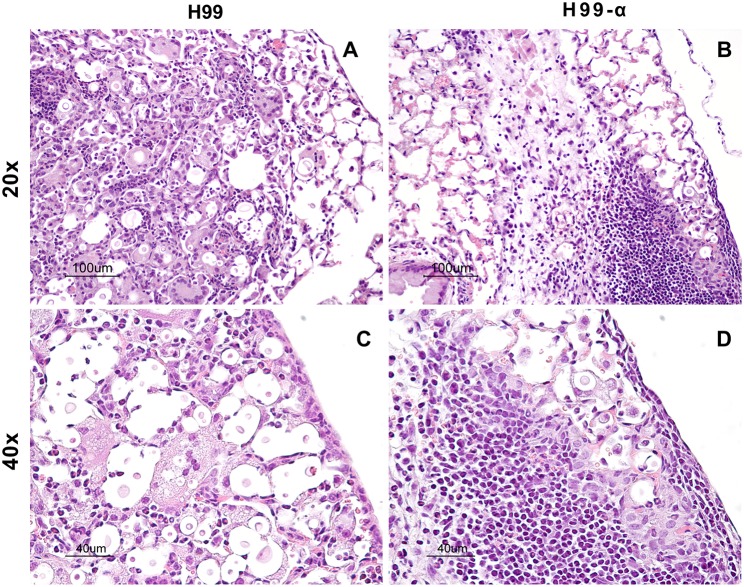
Production of TNF-α by the transgenic strain does not induce excessive inflammatory response or severe pathology in the infected lungs. Lungs from mice infected with H99-α or H99 were harvested at 14 dpi followed by Haemotoxylin & Eosin staining. The photographs were taken at ×20 **(A,B)** and ×40 objective power **(C,D)**. Note that H99-α-infected lungs showed different inflammatory features such as improved containment of the yeast, more condensed leukocytes infiltration around the infection site and less pronounced granulocyte responses than H99-infected lungs.

TNF-α is a potent pro-inflammatory mediator and the development of excessive inflammation could be a crucial concern while using TNF-α-based immunotherapy. Thus, our next goal was to determine the effect of TNF-α-producing strain on the extent of the inflammatory response and pathology in the lungs of infected mice. Our results supported that TNF-α induction by *C. neoformans* have not produced these undesirable effects. The lungs from H99 infected-mice showed a high level of inflammatory infiltrates at 14 dpi, however, most of the organisms within the alveolar space were not contained by the immune cells (Figures 4A,C). The cellular infiltrates contained predominantly cells with myeloid morphology (granulocytes and macrophages), consistent with previous reports. In contrast, H99-α infected mice showed nice compartmentalization of the infected and uninfected areas. Most parts of the lung tissues remained healthy and “clean” in comparison with the H99-infected mice. The fungal organisms were all contained within a confined area surrounded by dense inflammatory infiltrates, mostly lymphocytes and mononuclear cells (Figures 4B,D), consistent with improved host defenses in H99-α-infected group. No evidence of uncontrolled inflammation or inflammatory lung damage has been noted for up to 3 weeks of infection. These results demonstrate that the transgenic strain H99-α induces more focal and effective response compared to strain H99, which limits lung pathology at the corresponding time points.

### H99-α-Infected Mice Showed Selectively Increased Frequencies of T Cells and the Decreased Frequency of Eosinophils in the Infected Lungs Compared to H99-Infected Mice

**Figure 5:**
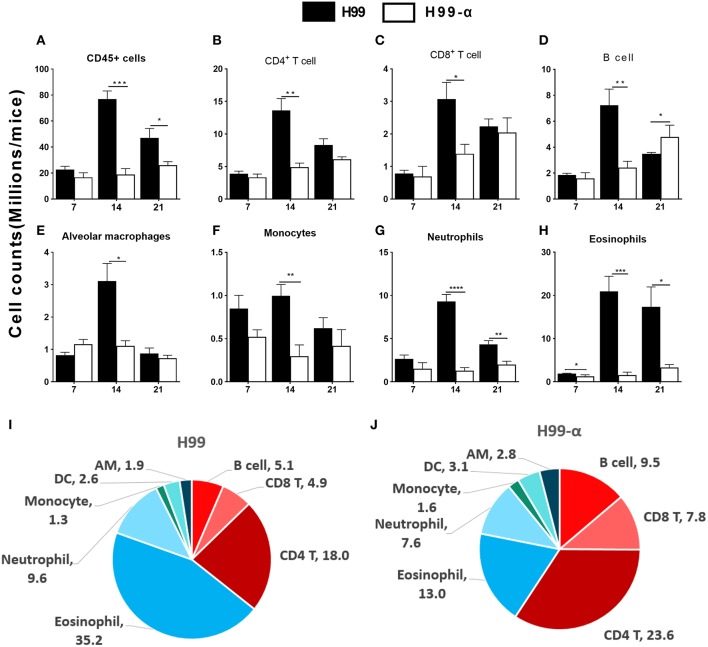
Production of TNF-α by the transgenic strain demonstrated a higher frequency of lymphocyte population than WT H99 stain. The subsets of immune cells in the infected lungs were analyzed by flow cytometry. CD45^+^ cell counts were calculated as total inflammatory infiltrates in the lungs after infection **(A)**. Cell number of CD4^+^ T cell **(B)**, CD8^+^ T cell **(C)**, B cell numbers **(D)**, alveolar macrophages **(E)**, monocytes **(F)**, neutrophils **(G)**, and eosinophils **(H)** were compared between H99-α and H99 infected mice. Frequencies (% of the CD45^+^ cells) of myeloid and lymphoid cell subsets in H99 **(I)** and H99-α **(J)** infected lungs at 21 dpi. Note that H99-α infected mice showed decreased leukocyte number but enhanced overall T cell and B cell frequencies in comparison with H99 group (Results represent mean ± SEM; ^*^P < 0.05; ^**^P < 0.01; ^***^P < 0.001; ^****^P < 0.0001 compared with H99 infected group at the same time point).

The histology results demonstrated that inflammatory recruitment pattern in the lungs infected with H99 and H99-α were different. To quantify cellular responses in the lungs infected with H99-α and H99, we isolated leukocytes from the dispersed lungs and conducted flow cytometry analysis. We found a reduced accumulation of total lung leukocyte numbers at 14 and 21 dpi in H99-α-infected mice (Figure 5A), which is consistent with our histology study. H99-α-infected mice showed the lower absolute numbers of CD4^+^ T cells, CD8^+^ T cells, B cells, alveolar macrophages, and monocytes compared to H99-infected mice, but more profound reductions were found in quantities of eosinophils and neutrophils (Figures 5B–H). We further found much higher frequencies of lymphoid cells of the adaptive immune system (CD4^+^ T cells, CD8^+^ T cells and B cells) in H99-α infected lungs compared to H99-infected lungs (Figures 5I,J). In contrast, H99 responses were dominated by myeloid cell populations, especially granulocytes (eosinophils and neutrophils) (Figures 5I,J). Collectively, these data show that H99-α skews the inflammatory response from myeloid cell-dominant toward lymphocyte-dominant pattern with a significant reduction in eosinophil accumulation.

### TNF-α Production by Transgenic *C. neoformans* Strain Shifts Cytokine Balance Away From Non-protective Type 2 Bias During Cryptococcal Infection

**Figure 6:**
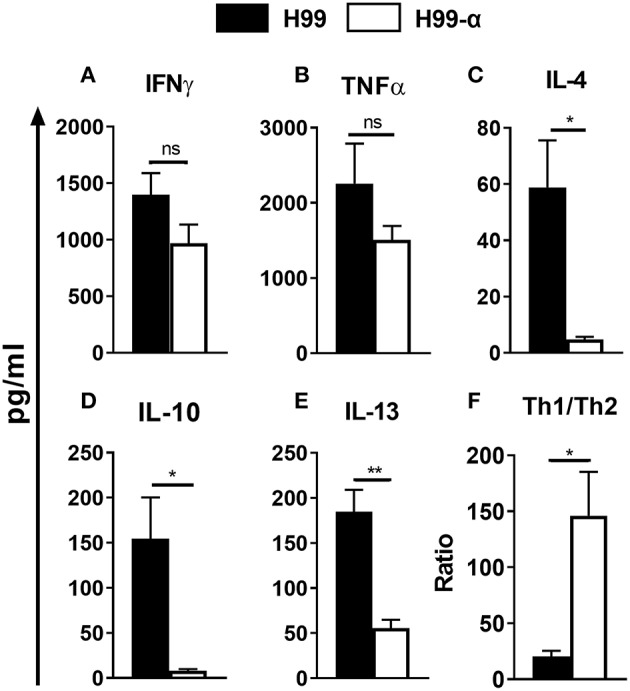
The TNF-α-producing transgenic strain opposed the development of pulmonary Th2 cytokine bias during cryptococcal infection. Total lung leukocytes were isolated on 14 dpi and incubated with heat-killed H99. The supernatant was collected followed by CBA assay. No significant difference was detected in IFN-γ **(A)**, and TNF-α **(B)** production. IL-4 **(C)**, IL-10 **(D)**, and IL-13 **(E)** secretion were profoundly attenuated in leukocytes from H99-α infected mice compared to WT H99-infected mice. **(F)** Th1/Th2 bias ratio was calculated as (IFN-γ+TNF-α)/(IL-4+IL-10+IL-13). Results represent mean ± SEM, *N* = 4 or more mice per group (^*^P < 0.05; ^**^P < 0.01; ns, no significant difference).

The changes in the cellular composition of the inflammatory cell infiltrate (notably, the reduced eosinophil accumulation) during H99-α infection suggested that production of TNF-α by the transgenic strain modulated Th1/Th2 bias and Th1/Th2-associated cytokine production in the lungs. We further analyzed the cryptococcal antigen-induced cytokine production by leukocytes from lungs at 14 dpi. Dispersed pulmonary leukocytes were adjusted to the same concentration, pulsed with the equivalent amount of heat-killed H99 and cultured for 24 h. Interestingly, lung leukocytes from H99-α and H99-infected mice showed a similar level of Th1 cytokines expression, including IFN-γ and TNF-α (Figures 6A,B), whereas expression of Th2 cytokines was profoundly diminished in H99-α-infected group compared with H99-infected group (Figures 6C–E). To assess if murine TNF-α expression by H99-α altered the overall Th1/Th2 balance, we evaluated the ratio of Th1/Th2-type cytokines production. As shown in Figure 6F, H99-α-infected mice significantly skewed the Th cytokine balance in a way that suggests more protective Th1 polarization, indicating a protective role of the TNF-α-producing transgenic *C. neoformans* strain in the efferent phase of infection. Finally, we performed qPCR analysis on adherence enriched macrophages from the infected lungs of H99-α and H99 infected mice (Figure S2). Interestingly, no significant difference was detected in IFN-γ or TNF-α expression; however, we found that the expression of IL-13 was significantly decreased in H99-α infected group compared to H99 infected group. Thus, the production of murine TNF-α by *C. neoformans* promoted protective altered cytokine profile shifting the balance form non-protective Th2 cytokine profile toward more protective.

### Production of TNF-α by the H99-α Transgenic Strain Enhances Activation of cDC1 and Ly6C^+^ Monocyte at the Efferent Phase of Cryptococcal Infection

**Figure 7:**
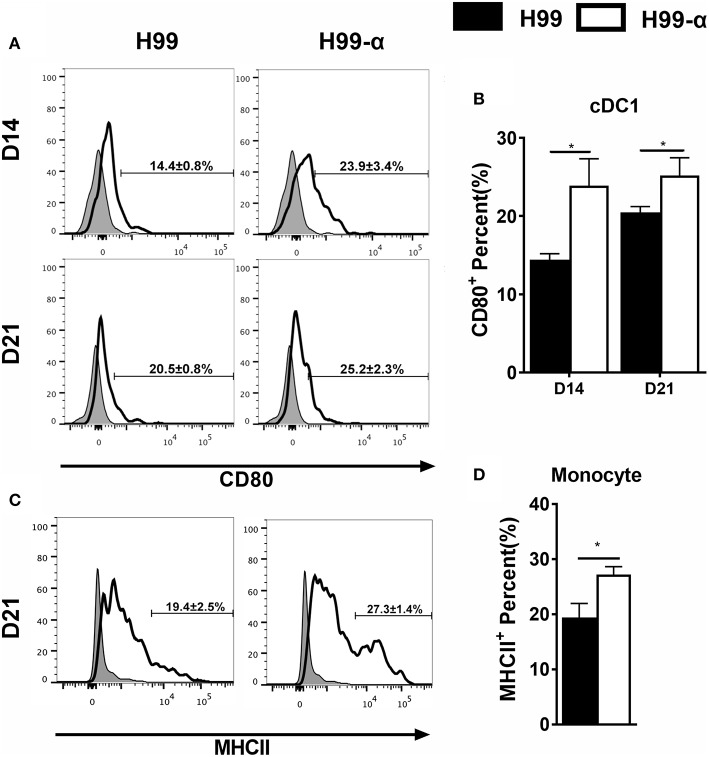
H99-α infected mice showed higher cDC1 and monocyte activation compared with H99 infected mice. Isolated lung leukocytes from infected mice were analyzed by flow cytometry. The surface expression levels of CD80 or MHCII were measured. **(A,B)** H99-α induced a higher frequency of cDC1 population producing CD80 on 14 and 21 dpi. **(C,D)** H99-α induced elevated frequency of monocytes producing MHCII at 21 dpi. Results represent mean ± SEM; *N* = 4 or more mice per group (^*^P < 0.05).

Having found that the H99-α prevented non-protective pulmonary type-2 cytokine bias, we asked whether this favorable shift in pulmonary immune responses was related to improved activation of lung antigen presenting cells. We analyzed monocyte and DC subsets: CD103^+^ cDC1, CD11b^+^ cDC2, as well as monocyte-derived DC (moDC) activation using CD80 and MHC class II molecule surface expression as readouts. H99-α infected mice showed elevated expression of CD80 in CD103^+^ cDC1 compared with H99 infected group at both 14 and 21 dpi (Figures 7A,B) and increased expression of MHC class II in monocytes subsets at 21 dpi compared with WT strain (Figures 7C,D). However, we did not observe any difference in activation in either CD11b+ cDC2 or moDC between H99-α and H99 infected mice (Figure S3). Collectively, our data demonstrate that TNF-α induced by H99-α may specifically enhance co-stimulatory activation of cDC1 and MHCII in monocyte during pulmonary cryptococcosis.

### Expression of TNF-α by the H99-α Transgenic Strain Diminishes Alternative Activation of Lung Macrophages and Promotes the Anti-cryptococcal Activity of Bone Marrow-Derived Macrophages in the Presence of IFN-γ

**Figure 8:**
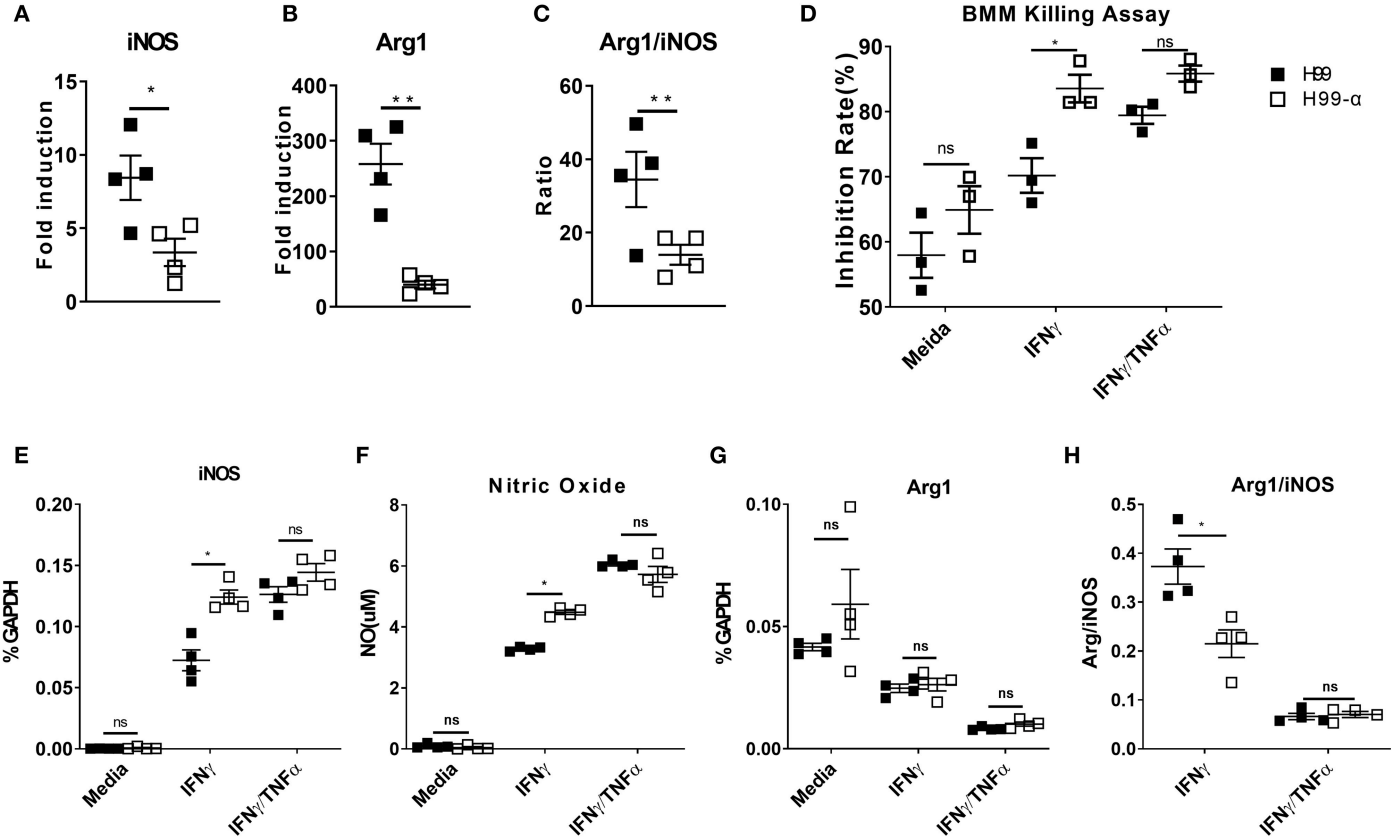
Expression of the murine TNF-α diminishes alternative activation of lung macrophages and promotes the anti-cryptococcal activity of BMMs *in vitro*. **(A,B)** mRNA expression of iNOS and Arginase (Arg1) from adherence-enriched macrophages at 14 dpi were determined by RT-qPCR. **(C)** Arg1/iNOS ratio was calculated as a readout of M2 vs. M1 activation bias. BMMs were co-cultured with mouse serum (10%) opsonized H99α or H99 in the medium only, IFN-γ, or IFN-γ together with TNF-α. Inhibition rates of fungal growth **(D)**, iNOS mRNA expression by BMM **(E)**, NO production in the supernatant **(F)**, Arg1 mRNA expression **(G)**, and Arg1/iNOS ratio **(H)** were evaluated. Notably, significantly higher growth inhibition rate, iNOS expression and NO production were found in macrophages challenged with H99-α than H99 in the presence of IFN-γ. Results represent mean ± SEM, *N* = 4 for each group (^*^P < 0.05; ^**^P < 0.01; ns, no significant difference).

Cytokine bias in the infected lungs directs macrophage activation patterns: classical activation (M1) leads to fugal containment and clearance, while alternative activation (M2) is non-protective and linked to progressive cryptococcosis (2). We thus examined the transcriptional expression of M1 hallmark gene nitric oxide synthase (iNOS) and M2 hallmark gene Arginase (Arg1) by adherence-enriched macrophages in the H99-α and H99 infected lungs. Arg1/iNOS ratio was also calculated as a readout of M2 vs. M1 bias. Macrophages from the H99-α infected lungs showed significantly lower expression of both *iNOS* and *ARG1* (Figures 8A,B), suggesting that both classical (M1) and alternative (M2) activation genes were upregulated to a lesser degree. This was consistent with significantly lower antigen load and less vigorous lung inflammatory response in the H99-α-infected group compared with H99-infected group. However, the Arg1/iNOS ratio was dramatically decreased in the H99-α-infected group relative to the control group (Figure 8C), suggesting that H99-α shifted overall macrophages polarization balance toward a less pronounced M2 activation.

While the observed changes in the adaptive immune response typically require time for the development of the immune response, our study showed significantly improved containment of H99a as early as 2 dpi. To determine if murine TNFα production directly affected macrophage fungicidal function, we examined *C. neoformans*, and bone marrow-derived macrophage (BMMs) interactions *in vitro*. Fungal growth inhibition and fungicidal nitric oxide (NO) production by macrophages challenged with H99-α or H99 were compared. Inhibition rates of fungal growth and iNOS mRNA expression were similar between macrophages challenged by H99-α or H99 in the absence of cytokine stimulation (Figure 8D). BMMs incubated with H99 or H99-α showed similar TNF-α mRNA expression (Figure S4). However, in the presence of IFN-γ, we observed significantly greater inhibition of *C. neoformans* by macrophages challenged with H99-α compared to macrophages challenged with H99. Consistently, we detected enhanced iNOS mRNA expression and nitric oxide production in macrophages challenged with H99-α compared to those challenged with H99 in the presence of IFN-γ stimulation (Figures 8E,F). While Arg1 expression level was similar between groups, the Arg1/iNOS ratio was significantly diminished in the H99-α infected group relative to the control group in the presence of IFN-γ stimulation (Figures 8G,H). The supplementation with exogenous TNF-α in addition to IFN-γ (TNF-α + IFN-γ BMM groups) resulted in most enhanced killing capacity and iNOS production by BMM that was no longer different in cells infected with H99 and H99-α (Figures 8D–H). These results demonstrate that TNF-α produced by H99-α, on its own, cannot improve the fungicidal function of macrophages, but it enhances these effects in the presence of IFN-γ activation to the similar degree as the exogenously added TNF-α.

## Discussion

The goal of this study was to enhance understanding of the role of TNF-α in anti-cryptococcal host defenses and to evaluate its supplementation as a strategy to enhance immunization against *C. neoformans*. To this end, we have engineered a *C. neoformans* H99-α strain capable of expressing murine TNF-α and explored its efficacy to stimulate protective anti-cryptococcal host responses. We demonstrate that mice infected with *C. neoformans* H99-α have vastly improved fungal containment in the infected lungs, showed limited lung pathology, resisted CNS dissemination, and gained a substantial survival benefit. These improved clinical outcomes were mechanistically linked to (1) the altered inflammatory responses in the lungs, with a selective increase in proportions of the adaptive immune cells (T cells and B-cells) and diminished non-protective characteristics such as eosinophilia; (2) significantly diminished non-protective Th2-type cytokine expression and thereby improved Th1/Th2 cytokine ratio; (3) and improved balance of M1/M2 macrophage polarization *in vivo* coupled with the improved *in vitro* fungicidal activity of primary macrophages against H99-α relative to H99, in the presence of IFN-γ stimulation.

Our studies found that the transgenic strain with TNF-α production enhances fungal containment by the host at the very early innate phase of infection. The growth of H99-α compared to H99 was significantly suppressed at 2 dpi and showed a strong trend for reduced fungal burden at day 1. This improved control of the transgenic strain is unlikely to result from attenuation of the strain itself following the integration of the TNF-α expression construct. We have found that the transgenic stain showed similar viability and growth rate at both 30°C and 37°C, unaltered expression of urease, laccase (melanin) and capsule, and similar sensitivity to the oxidative, high salt and osmotic stresses compared to the WT strain H99. Instead, this rapid suppression of fungal CFU strongly suggests that TNF-α production by H99-α enhanced the fungicidal function of the resident cells (macrophages and DC) since these effects became apparent even prior to the expected wave of leukocyte recruitment. The *in vitro* results also support that the effect of fungal-derived TNF-α on the fungicidal function of macrophages can be detected as early as 24 h, provided that an additional activating signal such as IFN-γ is present. Overall, these findings support that TNF-α expression by H99-α effectively activates the innate defenses to substantially reduce fungal burden right at the onset of the infection.

While the effects of TNF-α at the early defenses were insufficient to eliminate the infection with H99-α during the innate-phase, additional benefits of TNF-α production were observed during the adaptive phase of infection. Cell-mediated immunity, especially the involvement of Th1-type CD4 T cell, is the most effective host defense mechanism against *C. neoformans* infection in the lungs (2). Robust early TNF-α induction during *C. neoformans* infection stabilizes Th1 and prevent the development of non-protective Th2 (16, 17, 33). Consistently, we found that mice infected with TNF-α-producing strain showed remarkable alleviation in fungal expansion and the absence of Th2-driven lung tissue pathology in opposition to uncontrolled growth and rapid CNS dissemination of the WT H99 during the adaptive phase of host response, which is typically developed by 14 dpi (2, 13). In addition to the overall weaker inflammatory infiltration (most likely associated with much lower antigen load), H99-α shifted the immune response balance toward Th1, which is evidenced by (1) condensed immune cells infiltration around infection sites with restrained fungal dissemination; (2) altered inflammatory infiltration phenotype from eosinophil and myeloid cell-dominant toward lymphocyte-dominant pattern; and (3) the improved Th1/Th2 cytokine ratio.

The significant role of TNF-α in the generation of protective Th1 response in cryptococcal pneumonia have been demonstrated in several models. Most recently, we have reported that early TNF-α signaling plays a critical role in classical DC activation and subsequent Th1/Th17 immune responses in CBA/J mice infected with a moderately virulent strain *C. neoformans* 52D (34). Furthermore, delivery of a TNF-α-expressing adenoviral vector to C57BL/6 mice infected with *C. neoformans* 52D prevented Th2 development and promoted protective Th1 responses during cryptococcal infection, which was associated with improved pulmonary DC MHCII-maturation (35). In the present study, we have used a highly virulent strain H99, which induces strong Th2 bias as a part of its virulence strategy (11, 13, 36, 37). In keeping with previous findings, direct TNF-α production by yeast strain modulated Th1/Th2 balance during infection, and drove the improved co-stimulatory activation of CD103+ cDC1, reported to be specialized in promoting Th1 response (38). However, the predominant effect of TNF-α production was significant ablation of the Th2-arm without a concurrent amplification of the pulmonary Th1 cytokine expression. Thus, despite the significant improvement in overall Th1/Th2 cytokine balance, the H99-α infected mice were unable to develop a robust protective response and resolve the infection. This is further supported by high lung and brain fungal burdens detected in H99-α infected mice comparable to WT infected mice at the time of death. Altogether, our data overwhelmingly suggests that the mortality of H99-α infected mice was a result of insufficient host's control of the fungal growth that occurred despite somewhat improved immune response. Thus, cryptococcal expression of TNF-α is unlikely to become an optimal tool to induce complete immunoprotection against *C. neoformans* infection, in contrast with findings obtained with IFN-γ-producing *C. neoformans* strain, H99-γ (24). Primary infection with H99-γ not only becomes completely resolved but it also confers protection against a secondary challenge with the WT H99 strain (24). This comparison indicates that TNF-α ranks below IFN-γ when it comes to its importance as a protective cytokine. In this respect, the importance of TNF-α appears to match that of IL-12, of which supplementation results in improved survival and fungal control but not a complete clearance (39). At the cellular level, this is illustrated by the requirement of IFN-γ to unmask the beneficial effect of TNF-α for macrophage fungicidal activity *in vitro*.

Together, our studies show that TNF-α itself produced by transgenic *C. neoformans* strain is not sufficient to confer complete protection against highly virulent strain H99, despite the fact that early TNF-α production is critical for the development of protective responses during cryptococcal infection (16). Although in the current study we have not addressed several limitations, such as TNF-α delivery in clinical settings, the effects of increased dose of TNF-α supplementation, as well as the potential side effects of TNF-α that could come with higher doses of H99-α strain, our study opens a possibility that H99-α could become a beneficial “enhancer” when used in combination with H99-γ for vaccination strategy. Future studies are needed to determine whether combined immunization with strains H99-α and H99-γ would result in further improvement of protective effects induced by H99-γ alone.

## Data Availability

All datasets generated for this study are included in the manuscript and/or the Supplementary Files.

## Ethics Statement

All experiments were approved by the Veterans Affairs Institutional Animal Care and Use Committee under protocol number 0512-025 and were performed in accordance with NIH guidelines and the Guide for the Care and Use of Laboratory Animals.

## Author Contributions

MO, WL, and WF designed and supervised the project. ZF, JX, and JY performed the experiments, collected, and analyzed data. JS and QX designed the vector. WP and RY contributed to data analysis. ZF, JX, and MO wrote the article.

### Conflict of Interest Statement

The authors declare that the research was conducted in the absence of any commercial or financial relationships that could be construed as a potential conflict of interest.
