# Supplementary material for: TNF-α-Producing Cryptococcus neoformans Exerts Protective Effects on Host Defenses in Murine Pulmonary Cryptococcosis
Source: Front Immunol. 2019 Jul 26;10:1725. doi: 10.3389/fimmu.2019.01725 (PMC6677034; doi:10.3389/fimmu.2019.01725)
Supplement: Supplementary file 1 [file Presentation_1.pdf]

Supplementary Figures

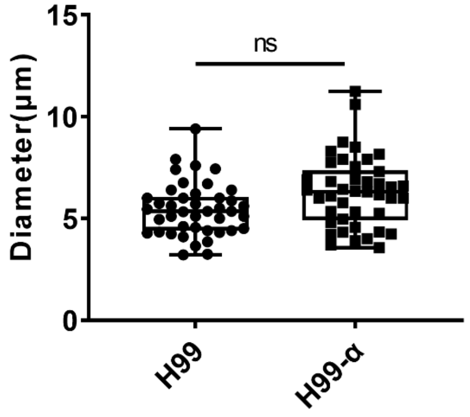

Figure S1. Capsule diameter of H99-α is comparable with WT *C. neoformans* strain H99. Cryptococcal stains were cultured in YPD broth until log phase. After another 72 hours-culture in DMEM under 37 °C and 5% CO_2, both strains were stained with India ink, and capsule diameters were measured under a microscope. N=50 for each group. (ns, no significant difference)

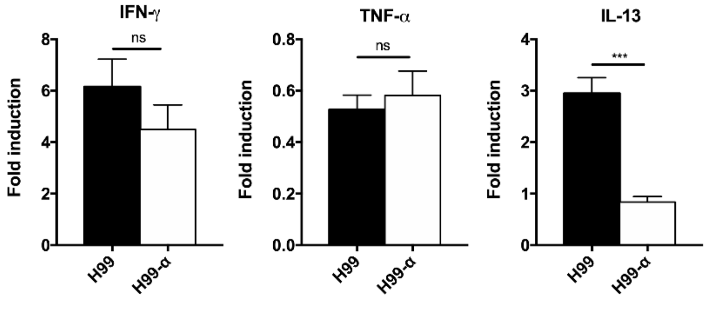

Figure S2. Expression of the murine TNF-α diminishes Th2 cytokine expression but not Th1 cytokines of lung macrophages. mRNA expression of TNF-α, IFN-γ & IL-13 from adherence-enriched macrophages at 14 dpi were determined by RT-qPCR. (***, $P < 0.001$; ns, no significant difference)

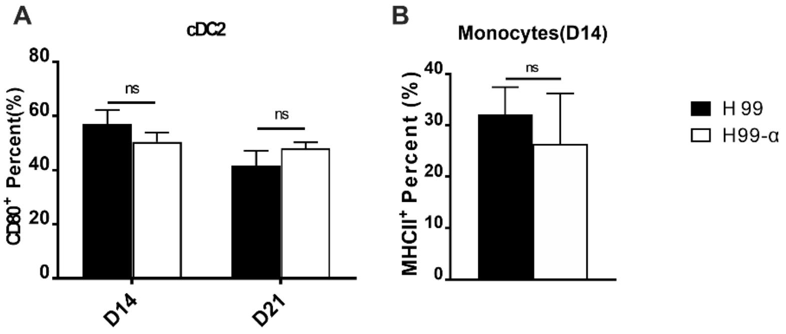

Figure S3. H99-α infected mice showed similar cDC2 activation compared with H99 infected mice. Isolated lung leukocytes from infected mice were analyzed by flow cytometry. The surface expression levels of CD80 or MHCII were measured. (A) Frequency of cDC2 population producing CD80 in H99-α infected mice is similar to H99 group at 14 and 21 dpi. (B) H99-α induced similar frequency of monocytes producing MHCII with H99 at 14 dpi. N=4 or more mice per group. (ns, no significant difference)

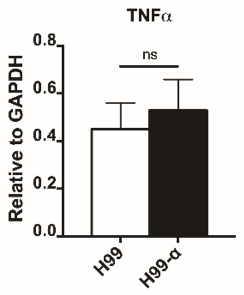

Fig S4 BMMs incubated with H99 or H99-α in vitro for 48 hours showed similar TNF-a mRNA expression. 1×10^5 BMMs were infected with H99 or H99-α (1×10^4) in 96-wells cell culture plate for 48 hours. RNA from BMMs was isolated, and TNF-α mRNA expression was evaluated by RT-qPCR. (ns, no significant difference)
